# A narrative review of the interconnection between pilot acute stress, startle, and surprise effects in the aviation context: Contribution of physiological measurements

**DOI:** 10.3389/fnrgo.2023.1059476

**Published:** 2023-02-23

**Authors:** Moussa Diarra, Mauro Marchitto, Marie-Christine Bressolle, Thierry Baccino, Véronique Drai-Zerbib

**Affiliations:** ^1^LEAD-CNRS, UMR5022, Université Bourgogne, Dijon, France; ^2^LUTIN, University Paris 8, Paris, France; ^3^Université Paris 8, Saint-Denis, France

**Keywords:** startle, surprise, stress, aviation, physiological measures, autonomic nervous system, sympathetic nervous system

## Abstract

Aviation remains one of the safest modes of transportation. However, an inappropriate response to an unexpected event can lead to flight incidents and accidents. Among several contributory factors, startle and surprise, which can lead to or exacerbate the pilot's state of stress, are often cited. Unlike stress, which has been the subject of much study in the context of driving and piloting, studies on startle and surprise are less numerous and these concepts are sometimes used interchangeably. Thus, the definitions of stress, startle, and surprise are reviewed, and related differences are put in evidence. Furthermore, it is proposed to distinguish these notions in the evaluation and to add physiological measures to subjective measures in their study. Indeed, Landman's theoretical model makes it possible to show the links between these concepts and studies using physiological parameters show that they would make it possible to disentangle the links between stress, startle and surprise in the context of aviation. Finally, we draw some perspectives to set up further studies focusing specifically on these concepts and their measurement.

## Introduction

While aviation remains the safest mode of transportation in the world, an inappropriate response to an unexpected event can lead to flight incidents and accidents. Some examples where an unexpected event led the pilot to make inappropriate/ineffective decisions leading to the crash are reported in the literature (Martin et al., 2015). Two factors, among others, are recognized as having played an important role in these events: **startle** and **surprise**. They were identified as determining events in many aviation issues. The analysis of incident reports in the Aviation Safety Reporting System (ASRS) from 1994 to 2013 shows 902 reports of surprise and 134 reports of startle. Among the incidents involving the startle, 37% involved a high intense stimulus (e.g., a loud noise) which interrupted the task in progress and/or provoked a protective reaction, while most of the incidents included an unexpected event or the absence of an expected event (Rivera et al., 2014). A more recent search on the Aviation Safety Reporting System (ASRS) database yields 4,361 incidents reports evoking “surprise” and 583 reports in which the term “startle” was mentioned (a wildcard % were used in the text search to imply all derivatives of these words, e.g., startl%, surpris%). For example, an instinctive reflex of Colgan Air Flight 3407 pilot (pulling back on the controls) following a startle event (a stall on an instrument-landing approach), is reported in the literature (Spangler and Park, 2010). These studies and reports have stimulated research on startle, surprise and stress.

In aviation, **startle** effect is defined as “an uncontrollable automatic muscle reflex, raised heart rate, blood pressure, etc., elicited by exposure to a sudden intense event that violates a pilot's expectations” [Federal Aviation Administration, 2015]. Similar to the definition given by the FAA, according to International Air Transport Association (IATA, 2018), startle is defined as “the initial short-term, involuntary physiological and cognitive reactions to an unexpected event that commence the normal human stress response”. The reflex motor response emanates from the brainstem (Groves et al., 1974; Bisdorff et al., 1994; Martin et al., 2015). In fact, “stimuli that are received through any modality (visual, auditory or tactile), are routed very rapidly to the amygdala, which makes a rapid appraisal of potential threat. It in turn sends signals to the top of the brain stem, where the reticularis pontis caudalis initiates motor responses *via* motor neurons emanating from the pons” (Martin et al., 2015, p. 98). In the presence of a threat, this first motor response is accompanied by an activation of a stress response within the autonomic nervous system (ANS), known as the “fight-or-flight” response. This physiological response is characterized by an increase in heart rate (HR), blood pressure (BP), and muscle contraction (Rivera et al., 2014). The startle response includes startle reflex as well as emotional (fear) and cognitive responses (attention deployment, interruption of ongoing activity). The startle response can be enhanced in magnitude following an aversive event or in threatening situations: this type of reaction is referred to as fear-potentiated startle, which have a longer-lasting effect and cause a fully developed stress reaction (Davis, 1993; Bradley et al., 2005; Eysenck et al., 2005; Martin et al., 2015).

Differently, **surprise** is an emotional and cognitive response to unexpected events that are (momentarily) difficult to explain, that force a person to change his/her understanding of the situation (Landman et al., 2017a). It could also involve higher level processes and activate frontoparietal cortical networks (Meindertsma et al., 2018). Indeed, fMRI studies indicate that surprise, unlike startle, would involve mainly subcortical brain regions, including the amygdala and striatum, as well as some cortical regions, such as the ventromedial prefrontal cortex and the cingulate cortex (Behrens et al., 2007; Bartra et al., 2013). Moreover, in a situation of persistent threat, this reaction leads to physiological responses related to those of stress [increased hearth rate, blood pressure (Bürki-Cohen, 2010; Rivera et al., 2014)]. Surprise is the consequence of a mismatch between mental expectations and perceptions of environment (Horstmann, 2006; Rivera et al., 2014). This suggests that the presence as well as the absence of a stimulus can elicit surprise. It contrasts with the startle effect, which is triggered by a sudden highly intensive stimulus and cannot be triggered by the absence of such a stimulus.

When startle and/or surprise effects are considerably important and persistent, they can lead to acute stress. Selye (1956) defined stress as “a response to change in order to maintain the state of stability or homology that the body has maintained against the stimulus to break the mental and physical balance and stability of the body” (Kim H.-G. et al., 2018). There are two kinds of stress: acute and chronic. Acute stress results from events involving novelty or a threat. The body reacts by releasing hormones that help to deal with the situation. Chronic stress results from repeated exposure to situations that lead to the release of stress hormones. There are close correspondences of ANS responses between acute and chronic stress, but also between an emotional reaction of surprise and the startle response, which is most often followed by an emotional response of fear (Bürki-Cohen, 2010; Coon and Mitterer, 2012). Many studies have been conducted to assess stress in pilots and/or in driving situations (Healey and Picard, 2005; Dehais et al., 2008; Tichon et al., 2014; Dismukes et al., 2015 for review; Alberdi et al., 2016). In their systematic review on acute physiological stress response to driving, Antoun et al. (2017) screened nearly 27,295 studies on the subject and selected 28 showing significant change in at least one physiological outcome.

However, not many studies compared to those on stress have specifically addressed the startle response and surprise effect in driving or flying situations (Thackray, 1988; Bürki-Cohen, 2010; Martin et al., 2012, 2015, 2016; Casner et al., 2013; Rivera et al., 2014; Schroeder et al., 2014; Ledegang and Groen, 2015; Talone et al., 2015; Landman et al., 2017a,b, 2018; Kinney and O'Hare, 2019; Xie et al., 2022). While it is clear that these two factors play an important role in the reporting of incidents and accidents (Talone et al., 2015), they are often used interchangeably in the aviation domain. More recently, Xie et al. (2022) suggested that startle and surprise place the pilot in a stressful state. These cognitive states (surprise, startle, stress) are intertwined and in the literature their definitions are similar and can be confounded.

For example, Rivera et al. (2014) definition of surprise “*Surprise is a cognitive-emotional response to something unexpected, which results from a mismatch between one's mental expectations and perceptions of one's environment*” can be confounded with Edwards and Cooper's (1988) definition of stress “*stress as a negative discrepancy between an individual's perceived state and desired state, provided that the presence of this discrepancy is considered important by the individual*”.

Results presented in the literature suggest that the startle response is strongly connected to the fight or flight response. Thus, Sehlström et al. (2022) show that physiological stress followed startling stimuli and Xie et al. (2022) mention that sympathetic nervous system weakness following startle, which manifests as a cognitive effect (attention shift), is related to stress.

All these elements contribute to the complexity of distinguishing these different cognitive states and to the fact that for example the terms startle and surprise are used interchangeably in the literature.

From a neurophysiological point of view, it has been shown that the amygdala and brain stem are involved in the startle response as well as the activation of the “fight or flight” stress response system through the hypothalamic-pituitary-adrenocortical (HPA) axis of the sympathetic nervous system (Martin et al., 2015). It should be noted that acute stress response or fight-flight response originates in the amygdala which sends a distress signal to the hypothalamus. It seems that startle and stress activate the same brain regions involved more generally in the response to threat. Concerning surprise, it has also been shown that the amygdala is involved in the response to a surprising event (Holand et al., 1999; Kim et al., 2017). Therefore, these three cognitive states are interconnected because they share a common brain structure or structures, which further contributes to the complexity of distinguishing them. It seems necessary to review their respective definitions, their differences and similarities and the methods used to study them in order to take them into account in their evaluation and the development of experimental protocols in the field of aviation. The presentation of the concepts is done in a distinct way in this paper in order to better highlight the differences but the reader is invited to take into account the fact that these concepts are interconnected and their distinction in real situation can be difficult.

The objective of this review is thus to better clarify the definitions of these concepts that are used and even manipulated interchangeably in the literature. From the theoretical model presented by the Landman et al. (2017a), it is shown the links and interconnections that these concepts share. This also participates in the complexity of distinguishing them and the ambiguity of what is actually manipulated in the studies present in the literature. Finally, emphasis is placed on the need to disentangle them as they are identified as key events in many aviation problems. And for this, the use of physiological parameters can be a real contribution to their studies and measurements. The contribution of this paper is to review the literature of experimental studies conducted on these concepts of startle, surprise, stress in aviation, to show their contributions and limits and the positive impact that the addition of physiological measures can have in their study in order to better distinguish them but also to reveal the links they maintain. This review is therefore structured along the following lines: (1) the methodology adopted by the review, (2) the definitions of startle, surprise, and stress as well as the difference between these cognitive states according to the conceptual model provided by Landman et al. (2017a) represented in Figures 2, 3 the studies that have been carried out. For each of the concepts studied (startle, surprise, stress), we also describe the physiological effects, the implications at the cognitive level, and finally suggest further investigations on some still open issues.

## Methodology

The review begins by searching the relevant papers that investigate surprise, startle and stress in the context of aviation. For this the searching strings included the terms “startle surprise stress” combined (“AND”) with one or more of the terms “aviation, pilot, airplane, aircraft”. The search is repeated in the following academic databases: Pubmed, Google Scholar, PsycINFO, IEEE Xplore and Elsevier.

The initial search results are then refined by the title, abstract and the objective of the study. As a general inclusion criteria, research articles evaluating startle, surprise or stress in aviation context are included. Since reviews on stress in aviation context exist in the literature, the emphasis was put on studies evaluating startle and/or surprise as a priority since these are less numerous. The rationale for this is that these terms are used interchangeably in the literature, they interact with stress, and the interconnections between them need to be unraveled.

## Startle effect

In the scientific literature, startle effect is defined as a brief, fast and highly physiological reaction to an unexpected, sudden, intense, or threatening stimulus such as a loud noise, or a sudden loss of balance (Thackray, 1988; Rivera et al., 2014; Martin et al., 2015). Following a startle event, the measurable effects are eye blinks (eyelid-closure), increased physiological arousal, stopping movements (freezing and/or reduced motor activity, Plappert et al., 1993), muscle tension (in particular contraction of the facial and neck muscles), and declarative reports of fear or anger (Landman et al., 2017a). The physiological responses induced by the startle effect involve physiological elements of survival in the limbic part of the brain (amygdala) and trigger an activation of the sympathetic nervous system (SNS) by the secretion of adrenaline and cortisol (known as the body's stress hormones). This automatic behavior induces a cognitive deterioration, such as information processing impairment between 30 and 60 s after the event occurrence (startle refractory period) (Vlasak, 1969; Woodhead, 1969; Thackray and Touchstone, 1970; Maslovat et al., 2015). It was studied in aviation because many induced cognitive disturbances negatively influence decision-making and problem-solving abilities, thus potentially leading to tragic events. Following a startle stimulus, the startle reflex occurs very quickly, i.e., about 14 ms after the event (startle-induced reflex movement in facial muscles, Yeomans and Frankland, 1995), and the performance at basic motor responses are disrupted for 3 s, up to 10 s for complex motor tasks (Rivera et al., 2014).

**Figure 1:**
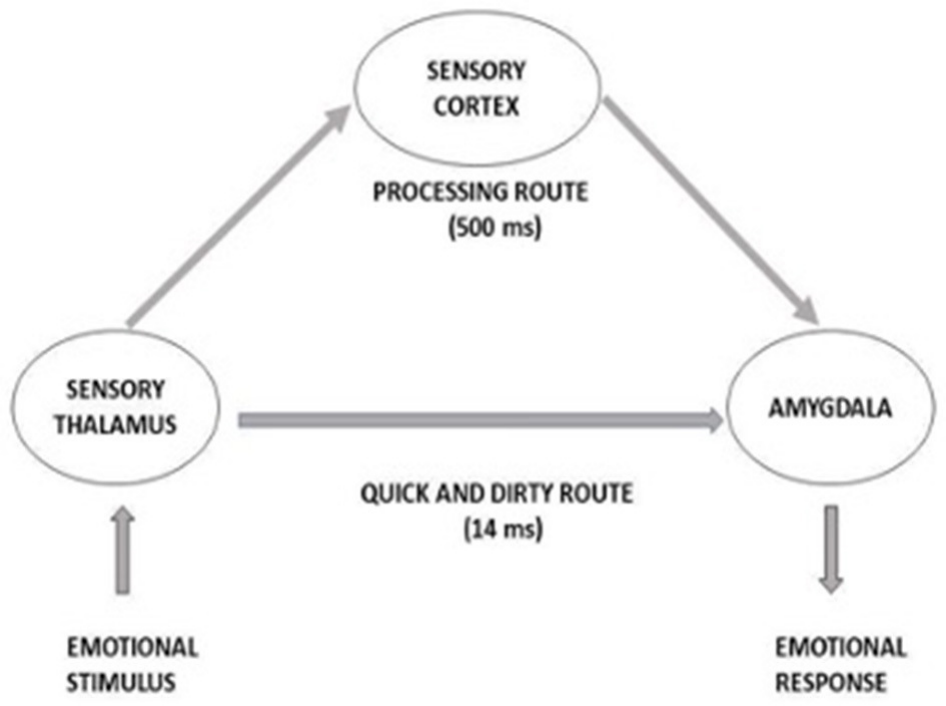
Two-ways processing of sudden stimulus eliciting startle effect. From Martin et al. (2012) adapted from LeDoux (1996).

Attentional resources orientation toward the startling stimulus is as well as cognitive response that, when exacerbated, can lead to decisional errors (Driskell and Salas, 1996). Several studies have determined the neural networks involved (Davis, 1993; Lindner et al., 2015). Thus, the amygdala center of emotions appears crucial to the startle response. While a signal with some significance induces the startle reflex, the same signal is sent to the sensory cortex for cognitive processing. While the processing in the prefrontal cortex takes around 500 ms (Åsli and Flaten, 2012), the amygdala analysis is very fast, leading to a startle reflex response (Figure 1). Cortical processing later returns to the amygdala to strengthen or modify salient information. This double treatment creates a gap between a spontaneous evaluation of the emotional valence of a stimulus by the amygdala and the cortical evaluation which can lead to a false alarm. When the threat persists, the startle moves from a simple reflex aversive movement to a startle or surprise reaction. This reaction leads to activation of the SNS and the endocrine system, and it is known as the “fight or flight” reaction. It affects also HR and BP by directing blood away from the extremities to the major muscle groups. This contributes for example to the state of confusion, delays in processing and/or attentional tunneling noted following a strong startle.

The physiological response to a startle event is attributed to the sympathetic response triggered by the emotional center, the amygdala. The release of norepinephrine (noradrenaline) on beta-adrenergic receptors, located on the sinus node and the ventricles of the heart, causes an increase in HR. The SNS includes the trochlear, oculomotor, and abducens cranial nerves, which innervate the muscles responsible for oculomotor movements. Thus, an amplified sympathetic innervation causes an increase in muscle contraction measurable at the intensity and speed of oculomotor movements. It also controls mydriasis (pupil dilation) used to improve the field of vision in an emergency. Following a startle event (e.g., a loud noise, an uncommanded lurch of the aircraft), an individual undergoes physiological arousal, and experiences an emotional state akin to fear (Dismukes et al., 2015). Thus, fear is a measurable aspect of startle. It should be noted that few aviation studies (Thackray, 1988; Kinney and O'Hare, 2019) have used physiological measurements to study the startle effect.

To sum up, during a startle there are three processes that occur over time: first, the startle reflex, which is an aversive physical reaction, is initiated very rapidly; second, the fight-or-flight reaction prepares the body for action by raising HR (tachycardia), and BP and introducing hormones such as adrenaline into the bloodstream. Third, a general activation of the SNS occurs in a wide-ranging stress reaction. False alarm startles will fade very quickly as cortical processing of the startling stimulus identifies the lack of threat: extinction signals will quickly replace activating signals. However, when an authentic threat is identified, the stress response will continue to develop. Research on fear conditioning has shown that when startle or surprise occur in the presence of perceived threat, the response can be exacerbated, leading to what is known as fear-potentiated startle, also referred to as acute stress (Kinney and O'Hare, 2019).

## Surprise

In addition to the startle effect, surprise is one of the factors that significantly contributes to inappropriate responses from the piloting team and to loss of control in flight (Horstmann, 2006). Surprise is a cognitive-emotional response to unexpectedness resulting from a disparity between expectations and actual perceptions (Rivera et al., 2014; Foster and Keane, 2015). Surprise can occur in the absence of startle, for example, when an event is appraised as not threatening at first sight and which evolves slowly. While the startle effect is an uncontrollable automatic reflex (involving muscles, HR, and BP) that is elicited by exposure to a threatening event, surprise too can be a slow emotional and cognitive response that stimulates research and a change in understanding of the situation. Research in this field has been done mainly at the level of mental schema theory. Schema (Bartlett and Bartlett, 1995) are knowledge structures stored in the long-term memory that represent “our” knowledge of the world and are used to recognize rapidly already known situations, but also to detect changing environment. Meyer et al. (1991) described surprise as a mismatch between activated schema and actual perception.

Surprise has a cognitive-emotional response to a stimulus similar to startle reaction, as for example fear and physiological responses such as increased HR and BP. Surprise cognitive responses include confusion, loss of situational awareness, interruption of ongoing task (freezing), inability to analyze and remember appropriate operating procedures (Bürki-Cohen, 2010; Rivera et al., 2014). Although surprise and startle cognitive states often take place together, they can also be experienced alone. Thus, the startle effect can occur without surprise, for example when the situation is anticipated, an individual is warned of the occurrence of a loud noise that does not prevent him from having a startle response (Ekman et al., 1985). Similarly, surprise can happen without startle, for example in aviation when subtle technical failures of automation occur, generating surprises that are ≪confusing≫ and difficult to explain.

Studies have shown that surprise occurs quite frequently in aviation, but in most cases, it is inconsequential for the outcome of the flight (Kochan et al., 2004). In extreme cases, surprise can impair the pilot troubleshooting capabilities.

The response time to a surprising event is longer than to a startle response. Indeed, the discrepancy between the current situation and the expected one forces to reassess the situation. The wider the discrepancy, the longer the time needed to reassess the situation. In addition, the surprise state lasts longer when the discrepancy requires an update of the surprised person's expectations (Horstmann, 2006).

Surprise increases arousal and draws attention to the triggering event. It mobilizes the attentional system on the most salient information, which is not the most important in that moment. This condition can significantly affect decision-making, problem solving, and critical skills in handling complex emergency situations. This allocation of attention toward the cause is known in research on emotions. Indeed, it has been shown that emotions modulate our perception and attention; in fact, the amygdala is involved in the processing of emotional information and has a direct impact on sensory cortices by increasing the neural representation of an emotional stimulus (Vuilleumier et al., 2004). However, it may also recruit fronto-parietal attention networks toward the location of the stimulus so that the information arriving at the same location as the emotional cues benefits from enhanced treatment resources (Brosch et al., 2013). Studies using fMRI have shown that the amygdala is most likely to be active at surprising situations, which can induce the activity of SNS (Holland and Gallagher, 2006; Kim et al., 2017).

Brainstem involvement is also reported for surprise, in addition to cortical and subcortical involvement (amygdala). Indeed, surprise can transiently boost arousal state, increasing the organism's sensitivity (Dayan and Yu, 2006) and suppresses ongoing beta-band oscillations within regions of prefrontal and parietal associative cortex (Meindertsma et al., 2018). These results are in line with the ideas that beta-oscillations help maintaining the current sensorimotor or cognitive state (termed the “status quo”) or help activating the currently relevant task set. In both frameworks, the need for maintaining the current status quo, or task set, is low in the case of surprise (Meindertsma et al., 2018). In addition, studies have shown an effect of surprise on the pupil dilation amplitudes which are closely linked to phasic responses in neuromodulatory brain systems, in particular the noradrenergic locus coeruleus (Kloosterman et al., 2015).

## Stress

Stress was defined as a maladaptive state in which SNS is overactivated, causing acute or chronic physical, psychological, and behavioral impairment (Campkin, 2000). “Both startle and surprise may cause acute stress” (Landman et al., 2017a, p. 1165). Startle may increase stress very briefly and rapidly at first, and subsequent appraisal of the startling stimulus as threatening may cause a further increase in stress. Surprise may also cause stress, as it may pose, on the one hand, an increase in task demands to solve the situation and, on the other hand, a perceived decrease of available resources when one becomes aware of the inadequateness of the active frame. The function of stress is to facilitate recruitment of additional resources to respond to the threat. In the aviation domain, number of studies have shown that aspects of stress such as impaired top-down and increased stimulus-driven attentional control, emotions of fear and frustration, excessive physiological arousal, or performance rigidity, may also impair a pilot's cognitive and motor performance (Dismukes et al., 2015; Landman et al., 2017a). It is therefore necessary to understand the impact of stress on cognition and performance, particularly in high-risk systems such as aviation, medicine, the military, etc., in which threats are widespread and can lead to devastating consequences.

In the literature, many studies have looked at pilot stress and mainly used HR, heart rate variability (HRV), and cortisol saliva as metrics. Indeed, the physiological, biochemical, endocrine, metabolic and immunological indicators change under stress. Faced with a stressful event, the first reacting system is the SNS that controls the functioning of organs such as the heart, the vessels, the lungs, the digestive system, and the secretion of catecholamines by the adrenal medulla (adrenaline and noradrenaline) which are sent to those organs in order to modify their functioning (Selye, 1950). The secretion of catecholamines and the stimulation they generate within the organs as well as within the nervous centers provoke the following reactions: vasoconstriction of peripheral vessels to reserve blood flow to the main organs (heart, lungs, and brain); increased blood pressure and heart acceleration; acceleration of oxygen in organs and muscles; preferential muscles vascularization (Selye, 1950; Dismukes et al., 2015; Martin et al., 2015). In the face of a stressor, the adaptive response of the body is extremely fast, organized by the sympathetic system and the medullo-adrenal gland under the control of the central nervous system. This is a general activation with emotional reaction (Gu et al., 2019; Wang et al., 2020).

Although several studies have been carried out to study stress in the literature, few studies have focused on distinguishing the physiological effects associated with the concepts of acute stress, startle effect, and surprise. More specifically, the startle effect was studied earlier in the 1970s to understand the consequences of a brutal (loud sound) shock on pilots (Thackray and Touchstone, 1970); differently, the surprise effect was studied more recently in connection with the automation surprise and the occurrence of an unexpected event in the cockpit (Kochan et al., 2004).

**Figure 2:**
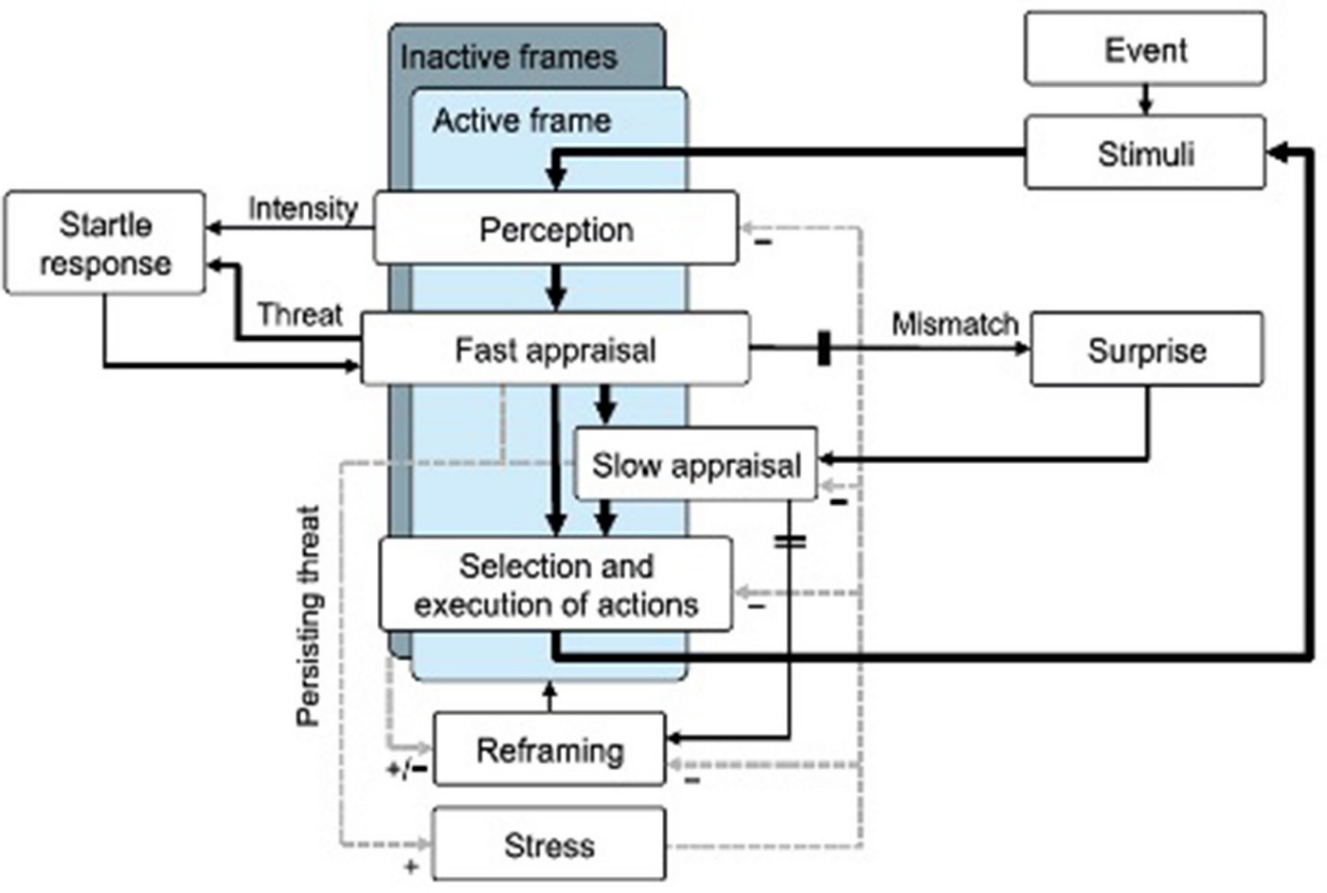
Landman et al. (2017a) conceptual model of startle and surprise. Solid lines indicate sequenced events. Dashed lines indicate potential influences, with plus signs indicating an increasing effect and minus signs indicating an impairing effect. Double lines indicate thresholds.

Landman et al. (2017a) recently proposed a conceptual model that brings together current knowledge about the startle effect, surprise and acute stress (Figure 2).

The authors present their model as a synthesis of sensemaking theories (Klein et al., 2007), cognitive models of surprise (Meyer et al., 1997), perceptual cycle model (Neisser, 1976) and literature on stress and the startle effect. In this model, the perceptual cycle is defined as the process by which a person perceives an external stimulus, interprets it (appraisal), assesses the situation, selects and executes actions. Appraisal can be fast and automatic but also slower and effortful. Decision making to action is a continuous process of hypotheses generation and test. In this model, the startle reaction results from a rapid and reflexive appraisal of a stimulus as threatening. When the threat is persistent, it can lead to stress increase. In the perceptual cycle, hypotheses based on the active frame are continuously tested and their consequences evaluated. The more the results are consistent with the hypotheses, the more the active frame is strengthened in memory. However, surprise is induced when there is a mismatch between the feedback and the active frame. Appraisal of surprising events is effortful and requires a reframing process that can lead to further increase of stress.

In this model, the startle reaction results from a rapid and reflexive appraisal of a stimulus as threatening. The left loop (*startle response*) is activated following a startle event without surprise. The appraisal of the situation and the response are extremely fast. Differently, when the appraisal of the startling situation highlights unexpected or incomprehensible information, the right loop (*surprise response*) is subsequently activated.

Surprise is described here as a mismatch between feedback from hypothesis testing of the active frame and their practical consequences (encountered data), given that the mismatch exceeds a certain threshold (double intersecting lines before surprise). Evaluation of a surprising event involves efforts (top-down or goal directed processing) to understand the cause of the mismatch between the data encountered and the active frame. It can be particularly problematic when pilots are not mentally prepared, for example after a long period of automatic flight (Young and Stanton, 2002).

Stress can impair slow appraisal and reframing, as these are more top-down or goal-directed processes, for instance by determining an incorrect frame selection, confusion, or loss of situational awareness. The authors of the model report that “stress is thought to cause a shift from analytical skills toward intuitive judgment, making one susceptible to biases. And this bias may, for instance, cause the incorrect application of a partially fitting frame that is easily retrieved from memory due to recent experiences” (Landman et al., 2017a, p. 1165). In fact, the adoption of an inappropriate frame or the loss of a fitting frame may lead to a complete “loss of grip” on the situation, because there is no longer a frame to guide perception and decision-making. This can negatively affect the pilot's ability to assess the situation and to make the right decisions, or lead to information overload (Landman et al., 2017a). In fact, the data encountered can no longer be compared to past experiences and they no longer make sense. Selection and execution of actions in this case become reactive and sequential (bottom-up) instead of being proactive and anticipatory (top-down). This can lead to a tunnel vision effect or even to a cognitive lockup (Sheridan, 1981; Landman et al., 2017a).

This conceptual model allows to provide elements to the design of experimental protocols in simulations inducing surprise or startle effect. Therefore, to induce surprise, they recommend setting up a situation that mismatches with a previously activated frame. A mismatch not immediately understood will incite to reframe the situation. Thus, the surprise and the reframing process can be shown by presenting well-known situations to the pilot and one subtly different. In order to induce a startle effect, a startle stimulus must be highly salient, as for example a loud and abrupt sound, or a sudden uncommanded motion of the aircraft. The magnitude of the accompanying ANS response will vary depending on the level of perceived threat. Then, the autonomic reaction can be substantial, cascading into a full stress response. Moreover, the startle stimulus will not require reframing the situation.

However, there are two major differences between the startle effect and the surprise. The startle results in an acute accentuation of stress that can lead to cognitive impairment and to reduced motor performance (Martin et al., 2015). Differently, surprise requires, before decision-making and action planning, an additional cognitive effort to reframe the situation and resolve the mismatch between current perception and previously activated patterns. This reframing process is effortful and vulnerable to the negative effects of stress that can potentially lead to confusion, inadequate frames adoption, and “loss of grip” on the situation (Landman et al., 2017a).

## State of the art on stress, startle and surprise experimental studies

While many studies have been carried out on stress in driving or flying situations (Kuroda et al., 1976; Lindqvist et al., 1983; Dehais et al., 2008; Iizuka et al., 2012; Regula et al., 2014; Tichon et al., 2014; Dismukes et al., 2015 for a review; Bruna et al., 2018; Cao et al., 2019; Shao et al., 2019), only few studies [mainly reports: technical (Field et al., 2015) and accident reports (Kochan et al., 2004; Rivera et al., 2014; Talone et al., 2015)] focused specifically on startle effect and surprise. Especially as mentioned by Rivera et al. (2014) and Landman et al. (2017a) most of the time these cognitive states (surprise, startle) are used interchangeably in the literature and no clear distinction is made between them. This makes it difficult to characterize each of them as well as the techniques and measures to assess them. Moreover, reports do not always specify explicitly whether the manipulation induced startle, surprise, or both, at least until the contribution by Landman et al. (2017a). For this reason, this section reviews this literature to introduce the methodologies adopted in such studies, trying as much as possible to group the studies according to the concept studied.

### Startle

Startle has been shown to negatively impact response time to simple and complex tasks. In a laboratory experiment (outside the aviation context) Sternbach (1960) showed that motor performance, i.e., key presses, is affected following an unexpected startle event (loud sound). The author tested the hypothesis of physiological differences between people (undergraduate students) who recover quickly from startle effect vs. those who recover slowly. Physiological measurements (cf. Appendix A for a synthesis of effects) showed that the latter had a greater increase in systolic blood pressure, pulse pressure, palmar skin conductance, HR and decrease in finger pulse volume. No difference was found for EEG measurements at frontal, temporal, parietal and occipital level. Similarly, May and Rice (1971) showed impaired motor performance and increased response time following a loud pistol shot in students and secretaries in a laboratory study. Alterations to simple reaction (1–3 s) and to information processing (30–60 s) after a startle stimulus have also been reported (Thackray, 1965; Thackray and Touchstone, 1983). Indeed, the authors compared the recovery performance following an auditory startling vs. non-startling event in simulated air traffic radar control. They note that their high intensity noise stimulus was manifestly startling while the lower intensity elicited only surprise reaction measured through physiological measures of HR, skin conductance, and video recording of the face. The results indicate longer response times and increased HR for the startled group. The participants in this group also made more errors in serial reaction time task. The authors note that recovery time for perceptual motor responses following an emergency shock takes 1–3 s, depending on whether the emergency was startling and emotionally arousing or only surprising and unexpected. If the shock causes emotional (and physiological) arousal as in a startle, then the information processing capacities can be altered for 30–60 s and lead to increased incorrect responses (Thackray, 1988). These studies suggest that the physiological reactions following a startle event are comparable to those following a surprising event, although of higher intensity for startle effect. These results support those of Vlasak (1969) and Woodhead (1969). The former showed impaired participant performances on continuous mental subtraction during the first 30 s following a startle stimulation (unexpected 100-db sound). The latter showed impaired performances on a continuous symbol-matching task lasting from 17 to 31 s after startle induced by a loud reproduced sonic bang in a laboratory context and with one hundred and eight Royal Naval ratings, which have acted as subjects. Startle effect has also a negative impact on ongoing cortical processes during go/no-go task (to press or not a button, depending on display color) in a laboratory experiment, with participants making more errors in startle vs. non-startle trials (Carlsen et al., 2008).

In aviation context, the results obtained previously in the laboratory are verified or amplified by the context. Bürki-Cohen (2010) defined startle as the ultimate surprising event with instant and uncontrollable motor responses, without distinguishing it from surprise as in the Landman model, in which the two responses might occur independently, with startle effect involving unexpectedness and intense stimulation, and surprise elicited by the presence or absence of an (un)expected event. Bürki-Cohen (2010) showed the interconnection between these two notions and mentions that depending on the pilot's anticipation, the same event can trigger either a surprise reaction or a startle, knowing that the latter is most often triggered by a unexpected event involving intense stimulation (e.g., compressor stalls, tire bursts, bird strikes, intense wakes). Moreover, it has been reported that the deterioration in pilot performance (cognitive impairment) is linked to the development of fear potentiated startle in response to an abnormal event (Rivera et al., 2014; Martin et al., 2015).

Few researches evaluated experimentally the impact of surprise and startle on pilot performance. Martin et al. (2016) designed an experiment to study the effects of a startle stimulus on pilot performance during abnormal events. This study is one of the few empirical studies explicitly studying the startle effect in pilots. Thus, they tested 18 pilots in a simulated flight involving two hand-flown instrument landing system approaches where the weather was such that a missed approach would have been required upon reaching the decision altitude. Startling stimulus was presented to the pilot through a cargo fire warning bell followed by a loud bang on the first approach. Following this, pilots were vectored for a second approach without startling stimulus. They were required to commence a standard missed approach when they failed to become visual at the minimum altitude. Height losses of second and first missed approach (i.e., following startle) were compared. The results indicate a deterioration of the performance for one third of the pilots following a startle, manifested by a delay in commencing the missed approach, or even some pilots continuing on an unstable approach up to affecting the safety of the flight. They also show interindividual differences in the startle response, some pilots more affected taking more time to recover compared to others less affected who recover quickly. No physiological measures are used to quantify the pilot's reaction as in previous studies evaluating the startle (Thackray, 1988; Martin et al., 2012, 2015; Rivera et al., 2014).

As suggested in the literature, it is highly recommended to measure physiological and subjective responses in order to account for the strength of a stressor (Bourne and Yaroush, 2003). Recently, Kinney and O'Hare (2019) extend Casner's studies and test the hypothesis that the startle effect can occur due to unexpectedness, without having to distract or cause a loud noise; they found that the startle response to the unexpected flight events (engine failure) contributes to the degradation of pilot performance. To this end, the authors use two physiological measures indicative of the startle response: HR and pupil diameter (PD). Twenty-two pilots faced a simulated expected or unexpected abnormal flight event (engine failure). In addition to physiological data, flight data (altitude, airspeed) and task performance were compared between these conditions. The results indicate that compared to an expected situation, pilots have a higher HR and increased PD when unexpected engine failure occurs. In terms of performance, 54.5% of pilots in the unexpected engine failure (with no forewarning) condition landed safely and 45.5% either crashed or attempted a water landing while no pilot had crashed in the expected condition. Authors also noted a reduction in instrument scan following an unexpected engine failure. These results are in line with previous studies showing the impact of an unexpected inflight event on performance and information processing (attention tunneling). It is noted that both experimental conditions lead to ANS arousal, more evident in the unexpected condition. If compared to other studies on the startle effect, the response to unexpected engine failure resulted in a similar arousal increase. Thus, it is possible to trigger a physiological response analogous to startle without any distracting or intrusive stimuli, by setting up unexpected events close to real life. According to Landman's model, it would appear that the unexpected condition was more startling due to the intensity or perceived threat.

The results of this study provide information on the usefulness of combining physiological and subjective measurements in order to verify the effects of the experimental manipulation on the subjects' responses. Comparison between expected, unexpected, and baseline flight showed that pilots' HR and pupil dilation were significantly different between the unexpected and expected engine failure conditions. Moreover, they were higher in the expected engine failure vs. baseline flight condition. This result confirms previous studies on the effects of surprise and startle. The surprise and startle reactions trigger similar physiological responses, but at different proportions, i.e., more sustained following a startling stimulus. Thus, HR and pupil dilation, which are widely used to measure acute stress, seem to be reliable indicators for future research on startle and surprise. Previous research on the effect of a startle response on HR indicates an increase from 7.5 to 15 bpm following a loud noise (Holand et al., 1999; Deuter et al., 2012; Chou et al., 2014). Kinney and O'Hare (2019) indicate an increase of 9.01 bpm following an unexpected engine failure.

More recently, a study by Xie et al. (2022) evaluated the effects of an abnormal flight environment (turbulence, startle, and surprise) using touch-based navigation displays (TNDs). They used Fitts' law, a predictive model used in human-computer interaction and ergonomics (Fitts, 1954), to compare the performance of TNDs with control display units (CDUs) and mode control panels (MCPs) under different flight scenarios (normal vs. abnormal). The authors suggested that startle and surprise can place the pilot in a stressful state. They noted that the startle had an influence on pilots' behavior by making them forget what to do. They simulated startle and surprise in their experience with failure scenarios (engine fire alarm) of the aviation system. Thus, they showed in their experiment that when excited by a startling and surprising scenario pilots are in a state of stress and prone to event interruption and the reaction time increased (Xie et al., 2022). They found that under normal flight conditions touchscreen interactive device show high accuracy and a short operation time. But under abnormal conditions, TNDs showed operation performance and stability worse than control CDUs and MCPs. In addition, under these conditions the pilots have less confidence in the touchscreen device. This study has the merit of distinguishing the notions of startle and surprise according to the theoretical framework of Landman et al. (2017a). However, the objective of this study was not to study the differences and/or interaction between these concepts, so the authors manipulated the two notions of startle and surprise together and not in a unitary way. An experimental setup manipulating, in addition, each of these notions could contribute further to a better understanding of the interaction and links between these mental states and stress. Nevertheless, as already mentioned, a link is made here between the surprise and the startle, which causes pilot stress. Performance and subjective measures are used in this study.

Rooseleer et al. (2022a) used neurophysiological measures coupled with subjective measures to measure pilot performance in severe wake turbulence events, which can induce the startle effect. The authors acknowledge that startle and surprise can have adverse consequences on pilot performance (inappropriate reaction, freezing, over reaction), and flight safety. Pilots are exposed to strong simulated wake vortex, with (wake imminent <1 mn & wake expected in 3 mn) or without prior ATC (Air Traffic Control) wake alert. The objective measures used by the authors are EDA, EEG & eyetracking data in order to measure pilot mental stress, workload and arousal and thus evaluate the impact of the wake vortex alert. The objective of this alert system is to reduce the startle effect that pilots may experience in case of aircraft upset induced by wake turbulence encounters. The authors state that the combination of psychophysiological measurements, expert observations and subjective feedback make it possible to better account for the performance of pilots with or without ATC wake alert as well as the potential advantages in terms of safety (reduction startle effect and potential loss of control Rooseleer et al., 2022b). And preliminary results show that the combination of subjective and objective measures make it possible to account for the effectiveness of the alert system “to prevent flight crew from startle response, smooth their workload and raised their situation awareness during the event” (Rooseleer et al., 2022b, p. 3149). It should be noted that the authors in order to study the startle effect used neurophysiological measures: EDA to evaluate the startle effect and EEG for stress as well as ocular data coupled with subjective measures.

In summary, the results from the literature have shown that the startle is physiologically measurable and is manifested by increase in blood pressure and heart rate (Rivera et al., 2014), eye blinks and pupil diameter modification, contraction of facial and neck muscles (Landman et al., 2017a; Kinney and O'Hare, 2019).

### Surprise

Beringer and Howard (1999) assess pilot responses to automation malfunctions and show maladaptive responses and longer reaction time response to situations that can lead to altitude loss or pilot disorientation. Casner et al. (2013) test eighteen pilots on three abnormal events: aerodynamic stall, low-level wind shear, and engine failure on take-off. This study, similar to that of Beringer and Howard (1999), evaluated pilot performance using response times, altitude loss, errors, pitch attitude, percentage of successful recoveries, and detection/correction times. No physiological measurements were used. The results indicate that response times are longer after a surprising event compared to a non-surprising event, and that correct performance of the procedures is impaired. Since unexpected events have been shown to generate startle effect (Ziperman and Smith, 1975; Kinney and O'Hare, 2019), the results of Casner et al. (2013) in terms of pilot performance impairment could be attributed to the effects of surprise and/or startle effect.

Schroeder et al. (2014) tested pilots on two stall maneuvers (high altitude vs. low altitude). In addition to the expert pilot rating, other performance measures based on flight data were used by the authors: maximum roll angle, altitude loss in the recovery, recovery time. Similar to the aforementioned studies, no physiological measurements were used in this study. A limitation that can be put in evidence in such a study is the absence of a control group to attribute the decline in performance to surprise. The authors conclude that the occurrence of an unexpected abnormal event in flight impairs pilot's response. Ledegang and Groen (2015) extend the previous results and observe that pilots are struggling in recovering from aerodynamic stalls when they have not reviewed the recovery procedures beforehand. In this study, the authors use flight parameters (time, altitude, angles control inputs, etc.) and subjective ratings as dependent variables. It is worth noting that no physiological measurement is used to assess the pilot's reaction to surprise.

Landman et al. (2017b) set up a study in a simulated environment in order to report specifically the impact of surprise on pilot performance. To overcome the limits of previous surprise studies, authors include a control condition (Schroeder et al., 2014) and a manipulation check (Casner et al., 2013). In addition to flight data and subjective ratings for surprise and startle, physiological measures are used to check the surprise manipulation. Recovery performance of twenty airlines pilots is tested in an expected (anticipated) and unexpected (surprise) stall event. In the last condition, experimenters adopt measures to mislead and distract the pilots into activating a cognitive frame that mismatches with the stall event. They were asked to pay attention to pitch because a spatial disorientation was possible and their attention was moved from the displays before the initiation of the event by asking them to give a rating on a sickness scale. The results indicate that only 75% of the pilots successfully adhere to standard recovery procedure in the surprise condition. Surprise negatively affected cognitive ability to manage an upset situation. Subjective and physiological measurements (HR, as well as Galvanic Skin Response, GSR) indicate that experience induces a greater proportion of surprise than of startle. These results are in line with the authors' model (Landman et al., 2017a), so it can be postulated that recovery was mentally more demanding in resources in a situation of surprise as compared to the anticipated situation, due to reframing process. As for subjective evaluation of surprise, startle, and mental load, significant differences between the two conditions were found for all of the three variables. Finally, as for physiological measurements, only galvanic skin response showed significant differences. However, a limit can be addressed to Landman et al. (2017b) study. Since studies have shown that distraction causes deterioration in flight performance (Barnes and Monan, 1990; Airbus, 2004; Bürki-Cohen, 2010), the performance impairment noted may be due either to surprise or to distraction. Nevertheless, this study fills the glaring lack of empirical research within aviation using physiological measurement.

In the above studies, the authors evaluated the subjective rating to the surprise and to the startle, although the surprise effect was manipulated in the first place. Their results suggest that the surprising upset event induced by their scenario was startling (Landman et al., 2017b). It is postulated that unexpectedness can trigger a startle response without the presence of intense stimulation. Indeed, it has been shown that unexpectedness can trigger surprise reaction (Rivera et al., 2014) and startle effect (Ziperman and Smith, 1975). However, there is a lack of physiological evidence to support those effects of startle and surprise in aviation.

The use of HR and GSR to evaluate the surprise effect by Landman et al. (2017b) also seem to be a good complement to subjective measurements. In their study, only the pilots' GSR response is significantly different between an expected (anticipated) vs. unexpected (surprise) stall events. The difference in HR, although not significant, shows an increase of 14 bpm for these two conditions compared to baseline. Indeed, pilot levels of perceived startle and surprise were collected on 11-points Likert-type scales and their ratings are significantly higher in the unexpected condition as compared to the expected condition. It is important to note that, in real-life situations, abnormal flight events likely induce startle and surprise, and therefore these factors are confounded in the pilot's stress reactions.

In summary, the results from the literature show that surprise can be studied physiologically and also manifests itself as for the startle by increased heart rate, increased blood pressure (Rivera et al., 2014) and finally increased galvanic skin response (Landman et al., 2017b).

### Stress

Bürki-Cohen (2010) notes that stress, surprise and startle are among the psychological factors involved in several accidents following a loss of control. As said above about the reactions to startle and surprise, the physiological and cognitive responses to surprise and stress are similar, although with different proportions. These reactions include increased HR, respiratory rates, BP, stress hormone secretion, sweating, change in GSR and narrowing of peripheral vision by pupil dilation (Bürki-Cohen, 2010). This author also notes that stress causes a loss of situation awareness and an oversight of adequate standard operating procedures applicable to the situation. Behaviorally, this can lead to a delay or absence of response (“freezing”).

As outlined in Landman conceptual model and recently by Xie et al. (2022), startle and surprise can both place the pilot in a stressful state. Thus, it is plausible to think that these two elements occur concurrently in real situations.

In fact, following startle, fight-or-flight reaction develops and SNS activates to prepare the body to react, i.e., both startle and surprise reactions cause acute stress, whose main role is to respond to emotional stressors which can disrupt the homeostatic balance. The typical response of SNS to a stressor is manifested by increased respiratory rate, heartbeat, blood flow to organs and release of catecholamines. These physiological changes are captured in research studies through various methods that can be used in the context of aviation in order to better account for and differentiate the concepts of surprise and startle, depending on the activation or inhibition of one of these indicators. In addition to these physiological measurements, there are measurements of brain activity. Indeed, studies have shown that the analysis of brain activity (e.g., EEG) makes it possible to account for the pilot's state of stress, so such measurements seem suitable (Fabre et al., 2021; Rooseleer et al., 2022a,b; Sciaraffa et al., 2022) for a better assessment of these in-flight cognitive states.

In summary, in order to account for the stress, startle and surprise effect in aviation, physiological measures (HR, EDA, oculomotor measures, muscle contraction, brain activity) can be proper measures to be coupled with subjective measures during experiments.

## Discussion and conclusion

The objective of the present paper was to review the definitions of stress, startle and surprise, the methods used to measure them. It stresses the real need to study the differences and interconnections that these concepts may have. As well as the fact that the analysis of physiological responses can contribute to disentangling differences between these cognitive states.

In aviation, few empirical studies have focused on the startle and surprise effect. Very few of them distinguish these two concepts, whereas the majority use them interchangeably. Nevertheless, accident reviews indicate a high proportion of causes related to surprise but also to performance following a startling stimulus.

In the literature, surprise most often refers to a cognitive-emotional response following an unexpected event, while the startle refers to an involuntary reflex following a sudden intense stimulation. Landman et al. (2017a) propose a conceptual model to distinguish the cognitive states of surprise, startle, and stress: the model indicates that they can occur independently as well as simultaneously. Based on both data from the literature and Landman's conceptual model, it appears that we could assess these cognitive states through the use of physiological measures, in addition to subjective measures.

Landman et al. (2017b) study showed that when surprise is manipulated in an experiment through an unexpected event, participants experienced a startling effect. And recently, Kinney and O'Hare (2019) showed that in order to trigger a physiological reaction similar to a startle effect, there is no need for a loud external stimulus, an unexpected threatening event might be enough. These results indicate the close links between these concepts. Biologically, the amygdala seems to be the most important hub that manages these two types of reactions. Neuroimaging studies have shown that surprise and fear emotions, noted following a startle, activate similar brain loci, such as amygdala and some studies suggest that surprise and fear might be the same basic emotions (Jack et al., 2014). Additionally, it has been proposed that surprise transiently boosts the central arousal state, mediated by phasic responses in brainstem neuromodulatory systems (such as the locus coeruleus noradrenaline system) (Dayan and Yu, 2006). Among the neurophysiological markers of locus coeruleus activity, studies have shown an increased pupil dilation (Rajkowski, 1993; Critchley et al., 2005), a greater P3 component of event-related potentials (Nieuwenhuis et al., 2005), and accompany salient action outcomes, such as errors. The involvement of locus coeruleus is reported when we are suddenly startled, or when we experience a stressful situation (Schwarz and Luo, 2015). It is also reported a relationship between an increased function of locus coeruleus and sympathetic activation; the greater the activation, the greater the correlation (Vermetten et al., 2002). Indeed, the norepinephrine from the locus coeruleus has an excitatory effect on most of the brain, mediating arousal and priming the brain's neurons to be activated by stimuli. It has also been reported that norepinephrine is the substrate for emotions that triggers “fight or flight” or acute stress response such as fear, and a monoamine model of basic emotions have been proposed (Wang et al., 2020). Furthermore, electrophysiological and neurochemical data have shown that brain norepinephrine system is activated by surprise/unexpected events (Ma and Morilak, 2005; Morilak et al., 2005; Bott-Flügel et al., 2011).

Coon and Mitterer (2012) considers surprise as a transient emotional state resulting from an unexpected event and which may have a different intensity (neutral, moderate, intense). Therefore, it is considered in some studies that experience with a surprising event commonly induces a startle response (Jang et al., 2015). Acute stress response or fight-flight response originates from the amygdala which sends a distress signal to the hypothalamus. Startle, surprise and acute stress are interconnected because they could share common brain structure or structures, which further contributes to the complexity of distinguishing them. Further studies will be needed to better identify measurable neurophysiological markers that could distinguish them.

In the model from Landman et al. (2017a), it can be noted that a startle reaction can be triggered by a stimulus according to its intensity or the perceived threat. Thus, an unexpected event could trigger either a surprise reaction or a startle response, depending on the intensity of the threat that it reflects for the subject. As a consequence, individual differences exist in relation to the reactions and the recovery following a startling, with some less affected and recovering faster than others (Sternbach, 1960; Martin et al., 2016).

Depending on the strength of the threatening stimulus, the physiological response to a startling and/or surprising event is similar to a stress response and measurable. Indeed, it has been shown that a persisting startle stimulus causes increase in HR and BP (Rivera et al., 2014), in addition to a motor response (muscle contraction). A startling event may also impact the respiration rate of pilots, although more evidence remains to be provided (Agha, 2020). In fact, there is evidence in the literature of startle modulation by respiration (Schulz et al., 2016). Bruna et al. (2018) showed an increase in the respiratory rate in pilots facing an unexpected engine failure during a simulated flight. Kinney and O'Hare (2019) suggested that an unexpected event can trigger a physiological response analogous to startle. Indeed, they showed that an unexpected engine failure changes PD and HR.

Surprise has also been shown to impact the physiological system measurable through an increase in HR and respiratory rate, GSR, PD (Landman et al., 2017b; Ruscio et al., 2017; Bruna et al., 2018; Kinney and O'Hare, 2019; Agha, 2020). Indeed, a persistence of the surprising/unexpected situation also increases stress.

Studies on stress have shown that it elicits several physiological responses: increased HR, respiratory rate, BP, muscle contraction, PD, ST, GSR, secretion of stress hormones (cortisol) (Lundberg et al., 2002; Healey and Picard, 2005; Otsuka et al., 2006; Kaklauskas et al., 2011; Dismukes et al., 2015) and brain responses (Alberdi et al., 2016; Kim H.-G. et al., 2018; Katmah et al., 2021 for a review).

Effects at the behavioral and cognitive level add to these physiological impacts. In fact, the startle reaction induces information processing impairment between 30 and 60 s (Vlasak, 1969; Woodhead, 1969; Thackray and Touchstone, 1970) which manifests behaviorally by a delay or absence of response (“freezing”), potentially leading to a deterioration of decision-making and problem-solving skills. A similar impairment of decision-making and problem-solving skills might also occur following a surprising event, since the attentional system is primarily mobilized on the most salient event, which often is not the most important information at that moment.

Finally, stress also impairs top-down cognitive functions and increases stimulus-driven attentional control. Indeed, Dismukes et al. (2015) noted that under stress, people are less able to manage their attention effectively, as they are more distracted from their task by the salience of the stimuli (alarm, threatening aspect). Therefore, they may process information less exhaustively, and they may have difficulty switching their attention among multiple tasks, thus resulting in chaotic situation management. In addition, individuals might have difficulty in making sense of the situation and in updating its mental model, thus making more errors. This is due to the fact that stressful/anxious thoughts tend to preempt working memory's limited storage capacity. Following a highly surprising/startling event, attention tunneling is another potential consequence of the experienced psychological stress. Importantly, stress influences attention as well as emotion. Indeed, it is typical to have psychological ratings that indicate a negatively valenced emotional stress response such as increased ratings of anxiety, irritability and loss of control. It is recognized that negative emotions such as fear and anxiety are cognitive stressors which have been shown to degrade decision making and situation awareness (Tichon et al., 2014). Indeed, the work of Weigmann and Shappell (1997) showed the impact of negative affects in many flight accidents, and Gluck and Gunzelmann (2013) underlined the important role of emotions in influencing cognitive processing and performance. Damasio (1995) also demonstrated the interdependent links between emotions and skills. Tichon et al. (2014) reported that emotions prepare us to respond to trigger stimuli by coordinating a system for responses. Thus, anger prepares the body to fight, and fear prepares it for flight (Matsumoto and Wilson, 2008).

In summary, the behavioral and cognitive consequences of stress, startle and surprise might be very similar. Therefore, measuring physiological metrics (besides subjective data) would help to quantify the proportion of physiological reaction to a certain event, thus enabling understanding the level of its impact (in terms of level and duration) on operational performance. It is important to conduct further research on these different concepts while controlling the confounding variables in order to better distinguish them. Because most studies in the field use the concepts of startle/surprise interchangeably. In order to account for the manipulations of each of these concepts and to better define the factors manipulated in the studies, it would be appropriate to add physiological and subjective data in order to better account for the state of an individual in a given experimental situation. The comparison of metrics such as HR, PD, GSR, could be used to account for the intensity of the stimuli and whether they were surprising or startling (or both) for the individual. Subjective measurements of related concepts like workload and stress should be used, in order to support conclusions made in relation to physiological data. Indeed, Tyler and Cushway (1995) have established that there is a significant and positive relation between stress level and workload.

More specifically, concerning the startle measurement, physiological measures such as the HR, HRV, change in GSR, in muscle contraction and in PD have been shown as effective, in addition to the traditional measures of reaction time and task performance. Other measures such as facial expressions are quite effective as it has been suggested that fear is a measurable aspect of startle (Dismukes et al., 2015). The optical system technique developed to remotely measure acoustic startle reflex (ASR) in humans also seems to be a promising technique (Balogiannis et al., 2020). Regarding surprise, evidence has been shown regarding the use of heart and respiratory rate, GSR, and pupil dilation. Time- and frequency-domain features of HRV and respiration appear to be additional measures. Similarly, Bruna et al. (2018) used respiration to assess stress. Moreover, various metrics have been shown to be valid for stress measurement: HR, HRV, EMG features (RMS), PD, Blink rate, change in GSR, hormonal secretion measurement (cortisol), BP, ST.

The results of the studies on startle effect and surprise stress the need to train pilots to unexpected events in order to better prepare them to react appropriately to real life unexpected events. Training to variable unexpected scenario in simulated environment could allow to extinguish fear-potentiated startle caused by unexpected situations encountered in flight situation (Landman et al., 2018). Moreover, the assessment of individual differences in response to unexpected events seems to be an important criterion in the selection and training of pilots.

Future research in the aviation field might be conducted based on the Landman model and using physiological measurements in order to study under what circumstances an unexpected event, e.g., an automation problem, can trigger a startle or surprise reaction and lead to a change in activated mental schema. Future research should shed more light on beyond what threshold of intensity or threat an abnormal event triggers a startle response. It would also be important to know what types of flight events are likely to lead either to an arousal indicative of a startle or to a surprise reaction among pilots. The effect of the pilots' expertise and experience should also be assessed. This research should be conducted in order to remove the ambiguity between these notions about measurement, therefore to define the type of stress reaction measured although similar to other stress type (stress following startle or surprise), avoid confounding factors (distraction), include control condition as well as manipulation check and larger sample size. These results could be used to inform other areas where performance and adherence to standard are impaired by unexpected events (nuclear power plant control, surgery, etc.) and provide good substrate for training.

In order to take into account the potential impact of these cognitive states on pilots, field studies in simulators could be considered in order to assess and confirm their physiological effects on the pilot. Thus, based on the presented experimental framework (Landman model + physiology) experimental manipulations evaluating one or more of these cognitive states according to the definitions and examples provided in this article but also those of the Landman model, coupled with physiological measurements having shown their sensitivity would make it possible to advance knowledge of the effects and measures of these cognitive states. This would allow to clarify their respective definitions, to avoid confusion in the terms used in the literature and in the reports of incidents and thus to provide training and countermeasures adapted to the negative impacts of these cognitive states. Neuroimaging techniques coupled with physiological measurements could also allow for the categorization of the structures involved in these states.

Thus, further experimental studies are needed to disentangle the close links that the notions of surprise, startle and stress may have. This will help to better understand the systems and structures involved in each of them and provide information on the best measure(s)/technique(s) to assess each concept.

## Author contributions

VD-Z, TB, and MD conceptualized the idea. MD performed the bibliographic search and wrote the first draft of the manuscript. All authors contributed to the revised manuscript, contributed to the article, and approved the submitted version.
