# Supplementary material for: A narrative review of the interconnection between pilot acute stress, startle, and surprise effects in the aviation context: Contribution of physiological measurements
Source: Front Neuroergon. 2023 Feb 23;4:1059476. doi: 10.3389/fnrgo.2023.1059476 (PMC10790839; doi:10.3389/fnrgo.2023.1059476)
Supplement: Supplementary file 1 [file Table_1.pdf]

Table 1: Techniques and dependent variables used to assess stress in the aviation context

Techniques	Concept studied	DVs	Results	References	Domain
ECG	Surprise/Startle	HR	increased heart rate	Kinney & O'Hare (2019)<br>Landman et al. (2017b)<br>Rivera et al. (2014)<br>Talone et al. (2015)<br>Burki-Cohen (2010)<br>Causse et al. (2012)<br>Lindqvist et al. (1983)<br>Lundberg et al. (2002)	Aviation
	Stress	HRV features	RR, RMSSD, pNN50, SDRR, HF & D2 decrease during stress<br>LF/HF, LF increased during stress	Castaldo et al. (2015)	Laboratory test (Stroop Colour Word, Academic examination, Arithmetical Task)
	Startle	HRV	pNN50, RMSSD decreased after acoustic stress	Jarczewski et al. (2019)	Laboratory test
	Stress	HRV	LF/HF increased	Regula et al. (2014)	Aviation
	Stress	HRV	SDNN, RMSSD decrease during stress, LF/HF increase	Cao et al. (2019)	Aviation

**Pupillometry/EDA/EMG**	Stress	PD/ Nonspecific Electrodermal Response Frequency (NS.EDR freq.) & EDA Area	PD increase during stressful situations; NS.EDR freq. did not and EDA seems to have less discriminating power compared to PD.	Pedrotti et al. (2014)	Driving automobile
	Stress	PD, blink rate, saccade rate fixation duration, Amplitude of EMG signal expressed as root-mean-square (RMS) value	PD, saccade rate and fixation duration correlate significantly with anxiety as self-report by participant. Blink rate non-significant. Muscle activation correlate positively with higher anxiety	Tichon et al. (2014a, 2014b)	Aviation
	Startle	PD	larger PD increase	Kinney & O’Hare (2019)	Aviation
**EMG/ECG/EDA/Respiration**	Stress	Amplitude of signal, root-mean-square (RMS) value, HR	Stress induced significant increases in muscle activity and in heart rate	Lundberg et al. (2002)	Laboratory test (mental arithmetic, Stroop color word)
	Stress	HR, HRV, Normalised mean & variance EMG/EDA /Respiration signal	EDA & HR are closely related to driver stress	Healey & Picard (2005)	Driving automobile
	Startle	Amplitude & frequency of signal	Startling acoustic stimuli induce muscle activation	Bisdorff et al. (1994)	Laboratory (free fall, acoustic startling stimuli)
	Stress	Amplitude/frequency of signal	Increase in EMG amplitude (RMS)/frequency (lower frequency) between stress and rest conditions.	Wijsman et al. (2010)	Laboratory (calculation task, logical puzzle task and a memory task)
	Stress	Mean, median, standard deviation of HR & respiration frequency	EMG & respiration signals are effective in stress level detection (97,8% & 86.7% of averaged correct detection rate respectively)	Wei (2013)	Laboratory task (subject playing puzzle game was put alternately in periods of high/low stress)

**Respiration/ECG/EDA**	Stress	Time/ frequency features for HRV & Respiration: Average of breathing frequency (ABF), average amplitude of minimum and maximum pairs in one breath cycle, standard deviation of amplitude of single breath cycle, Standard deviation of breathing frequency, respiration spectral power (RF1-RF4), Entropy measures.	Breathing parameters ABF, RF1, RF2, RF3, and RF4 increase significantly during stress phases.	Bruna et al. (2018)	Aviation
**EDA/ECG**	Surprise	HR/Phasic change in GSR Signal (Peak of skin conductance 10 s following upset -Peak of skin conductance 10 s before upset)	Surprise increases GSR in surprise vs anticipated condition	Landman et al. (2017b)	Aviation
**EEG/ECG/Salivary cortisol/EDA/EOG/Respiration**	Stress	HRV/EEG Beta frequency band/Cortisol levels	Salivary cortisol correlated negatively with HRV measure (SDNN). High stress group showed decreased HRV features. There was a significant negative correlation between SDNN and relative high beta power at both the anterior temporal sites. And positive correlation between cortisol and high beta activity.	Seo et al. (2010)	Laboratory (un/pleasant images induce stress)
	Stress emotions (surprise, scare, angry)	Mean, RMS, Standard deviation, variance, entropy of sample for HR/EEG (beta band)/GSR /HRV	Lowest recognition errors were reached when all data (EEG) were considered or when the GSR datasets were omitted from the training model	Roza & Postolache (2019)	Aviation

	Stress	Systolic/Diastolic blood pressure/HR	BP, HR & ST increase during stress and depending on the strength of the stress experienced	Kaklauskas et al. (2011)	Laboratory (Web-based Biometric Computer Mouse Advisory System to Analyze a User's Emotions and Work Productivity)
**BP/HR/ST/EEG/PD**	Stress	Mean, SD for Systolic/Diastolic blood pressure. HRV (HF, LF, LF/HF)	HF decrease & LF/HF increase during stress condition compared to control session. No difference for BP which increase during stress and control condition.	Hjortskov et al. (2004)	Laboratory (cognitive and emotional stressors during standardized computer task: lack of support, memory test)
	Stress/Startle	PD (saccade count, dwell time); Mean/standard deviation SBP, DBP, HR	PD is higher during stress condition vs neutral condition. Participants under stress visually sampled less in terms of both saccade count and dwell time on a cognitive impulsivity task (MFFT). No difference for BP.	Stankovic et al. (2014)	Laboratory (aversive tone stressor)
	Stress	SBP/DBP	SBP & DBP increase during flight and stressful flight segments	Melhado et al. (2000)	Aviation
	Stress	BVP (period, amplitude, LF/HF), GSR (Mean value, number of the responses and the areas under the responses), ST (Mean value ST), PD (Mean value)	Physiological signals correlate strongly with changes in emotional state of subjects when stress. PD was the most significant affective state indicator, compared to the other three physiological signals.	Zhai & Barreto (2006)	Laboratory (Stroop) Color-Word Interference Test)
**BVP/GSR/PD/ST/EDA**	Stress	Median HR/EDA/ST	HR demonstrated significance between stress and non-stress condition. Poorer stress discrimination accuracy for each metric	Rhudy et al. (2020)	Laboratory (International Affective Picture System)

	Stress	Mean, Standard deviation in time and frequency domain for HRV (0.05-0.5 Hz)/EMG (0–16 Hz). Latency, amplitude for EDA. Mean, min, max and standard deviation for ST.	Human stress identification using multiple physiological signals give good results with accuracy up to 93% for HRV signals, 76.25 %, 71.25 %, 70.32 % for ECG, EMG and ST signals respectively.	Palanisamy et al. (2013)	Laboratory (mental arithmetic task)
**ST/ECG/EMG /GSR**	Stress	The differential energy between forehead (average value) and philtrum (maximum value) (DEFP). Change rate of the HBR and cortisol level during the stress status.	Temperature increase during stress condition also HR and cortisol level. Extracted thermal imprints correlates with stress markers (heart beat rate and cortisol level)	Hong & Hong (2016)	Laboratory (Trier Social Stress Test)
**Thermal imaging/EEG/Cortisol/Alpha-amylase**	Stress	Forehead, Corrugator, Periorbital, Nose tip, Perioral, Chin, Finger Temperatures; HR, LF, HF, LF/HF, Alpha-amylase and cortisol level	Corrugator temperature, nose tip temperature, perioral temperature, chin temperature, heart rate, finger temperature, alpha-amylase and cortisol showed significant changes over time. Chin and finger temperatures decreased during stress, HR increased, Alpha-amylase & cortisol levels increased after stressor onset.	Engert et al. (2014)	Laboratory (Trier Social Stress Test)
	Stress	Cortisol level	Salivary cortisol level of student pilots increased post flight compared to preflight and were also greater compared to instructor pilots.	Otsuka et al. (2006)	Aviation
**Cortisol**	Stress	Salivary alpha amylase level	Alpha-amylase levels of four pilots at postflight were elevated compared to levels at preflight suggesting that tension is high during landing.	Iizuka et al. (2012)	Aviation

**Alpha amylase**	Emotional arousal	Happy, sad, angry, surprised, scared and disgust; HRV (Poincare plot indexes SD1, SD2), GSR amplitude	Landing and take-off present more variability or emotional events.	Roza & Postolache (2018)	Aviation
**Facial expressions/ECG/GSR/EEG/Questionnaires**	Stress	Fundamental frequency mode	increased during stressful situation	Wittels et al. (2002)	Real-life
**Speech analysis**	Stress	Features based on Teager energy operator & speech multiresolution analysis	Speech features allow to classify above chance level driver stress	Fernandez & Picard (2003)	Automobile
